# Annual acknowledgement of reviewers

**DOI:** 10.1186/1471-2431-13-30

**Published:** 2013-03-28

**Authors:** Emily Crow

**Affiliations:** 1 BioMed Central, 236 Grays Inn Rd, London WC1X 8HB, United Kingdom

## Abstract

**Contributing reviewers:**

The editors of *BMC Pediatrics* would like to thank all our reviewers who have contributed to the journal in Volume 12 (2012).

## 

Leif Edvard Aaroe

Norway

Elisabeth Adderson

USA

Monika Agarwal

India

Anju Aggarwal

India

A. J. Agopian

USA

Carlo Agostoni

Italy

Seyed-Moayed Alavian

Iran

David Alexander

Canada

Anna Alisi

Italy

Eman Alissa

Saudi Arabia

Naim Alkhouri

USA

Sanjiv Amin

USA

Howard Amital

United Kingdom

Generoso Andria

Italy

Michael Antunes

USA

Shmuel Arnon

Israel

Stephen Aronoff

USA

Marco Enrico Giovanni Arosio

Italy

Kamal Arya

India

Ambika Ashraf

USA

Paul Ashwood

USA

Susan Astley

USA

Elizabeth Asztalos

Canada

Inge Axelsson

Sweden

Denis Azzopardi

United Kingdom

Carl Backes

USA

David Bader

Israel

Egil Bakkeheim

Norway

Patrick Ball

Australia

Yaron Bar-Dayan

Israel

Stephen Barenkamp

USA

Lisa Barnett

Australia

Laura Basterfield

United Kingdom

Sriparna Basu

India

Jules Becher

Netherlands

David Becker

USA

Dean Beebe

USA

Jacob Ben Chaim

Israel

Jay Berry

USA

Jacquelyn Bertrand

USA

Michiel Betjes

Netherlands

Dominik Bettenworth

Germany

Julie Bettinger

Canada

Vineet Bhandari

USA

Nita Bhandari

India

Piyali Bhattacharya

India

Shilpa Bhupathiraju

USA

Sebastiano Bianca

Italy

Rosemarie Bigsby

USA

Ari Bitnun

Canada

Anders Bjerg

Sweden

Vivian Black

South Africa

Jonathan Blau

USA

Nem Yun Boo

Malaysia

Marcela Bottino

Brazil

Alex Bottle

United Kingdom

Luigi Bouchard

Canada

Pascal Bovet

Switzerland

Loretta Brabin

United Kingdom

Silvia Bressan

Italy

Beau Bruce

USA

Heather Brumberg

USA

Johan Nikolai Bruun

Norway

Monika Bullinger

Germany

Monica Burnett

USA

Gianfranco Butera

Italy

Davide Caimmi

Italy

Silvia Maria Elena Caimmi

Italy

John Cairney

Canada

Patrina Caldwell

Australia

Juan Antonio Camacho

Spain

Peter Camfield

USA

Kristine Campbell

USA

Marsha Campbell-Yeo

Canada

Michelle Cardel

USA

Teresa Cardesa

Spain

Waldemar Carlo

USA

Jorge Carneiro

Brazil

Norman Carr

United Kingdom

Julia Carroll

United Kingdom

Katia Castetbon

France

Marco Cattalini

Italy

Luigi Cattarossi

Italy

Francesca Cavataio

Italy

Mauro Ceccanti

Italy

Mustafa Celik

Turkey

Maja Cepanec

Croatia

Zeynep Cetin

Turkey

Ravichandran Chandrasekaran

India

Tai-Tsung Chang

Taiwan

Rima Chedid

Lebanon

Michael Chez

USA

Benjamin Chi

Zambia

Mohammod Chisti

Bangladesh

Catherine Chittleborough

Australia

Imti Choonara

United Kingdom

Catherine Choong

Australia

Urai Chotigeat

United Kingdom

George Chrousos

Greece

Charlene Chu

Canada

Albert Chudley

Canada

Tammy Chung

USA

Nursan Cinar

Turkey

Marco Cipolli

Italy

Olivier Claris

France

Tom Clarke

Ireland

Dylan Cliff

Australia

Harvey Coates

United Kingdom

Geraldine Coburn

Canada

Daniel Cohen

United Kingdom

Raanan Cohen-Kerem

Israel

Keith Collard

United Kingdom

Hannah Connell

United Kingdom

Adrian Cook

United Kingdom

Filip Cools

Belgium

Carrie Cox

USA

Margaret Creedon

USA

Chantal Cripe-Mamie

Switzerland

Magdalena Cuenca Garcia

Spain

Annet Dallmeijer

Netherlands

George Datto

USA

Michael Davidovitch

Israel

Frans De Baets

Belgium

Beatriz de Jongh

USA

Jose Ricardo de Mello Brandao

Canada

Giuseppe De Nisi

Italy

Carolina de Weerth

Netherlands

Andrea de Winter

Netherlands

Amanda Dempsey

USA

Eugene Dempsey

Ireland

Anne Demulder

Belgium

Sharon Demuth

USA

Johan Denollet

Netherlands

Amare Deribew

Ethiopia

Girish Deshpande

Australia

Antonio Dessanti

Italy

Ilker Devrim

Turkey

Maria Cristina Digilio

Italy

Gabriel Dimitriou

Greece

Constantine Djedjos

USA

Yvonne Dommels

Netherlands

Steven Donn

USA

Paulette Douek

Brazil

Benard Dreyer

USA

Marjan Drukker

Netherlands

Katrina Dubose

USA

Karel Duchen

Sweden

Jennifer Duchon

USA

Trevor Duke

Australia

Carlos Duran

Vanuatu

Sourabh Dutta

Canada

Kevin Dysart

USA

Tobias Edbom

Sweden

Deborah Ehrenthal

USA

Joey Eisenmann

USA

Gregory Elliott

USA

Pauline Emmett

United Kingdom

Kynan Eng

Switzerland

Susan Ennett

USA

Leif Eriksson

Sweden

Vanesa España Romero

USA

Günter Esser

Germany

Analee Etheredge

USA

Piero Farruggia

Italy

Melissa Faulkner

USA

Mohamed Eid Fawzy

Egypt

Janusz Feber

Canada

Emily Feinberg

USA

Michael Feldman

Israel

Tanis Fenton

Canada

Jennifer Fereday

Australia

Ricardo Fernandes

Portugal

Juan Miguel Fernandez-Alvira

Netherlands

James Fett

USA

Alessandro Fiocchi

Italy

Gordon Fisher

USA

Joao Borges Fortes Filho

Brazil

Stephanie Franchi-Abella

France

Luisa Galli

Italy

Kathleen Ganley

USA

Michael Ganz

USA

Lourdes Garcia

Puerto Rico

Hector H. Garcia

Peru

Sumit Gaur

India

Cindy Gauvreau

Canada

Mike Geraint

United Kingdom

James Gern

USA

Reinaldo Gianini

Brazil

Maddalena Giannella

Italy

Donzelli Gianpaolo

Italy

Francis Gilchrist

United Kingdom

Maria Teresa Giner

Spain

Frances Glascoe

USA

Ronald Gold

Canada

Borja Gomez

Spain

Luis Gracia-Marco

United Kingdom

Mats Granlund

Sweden

Cori Green

USA

David Greenberg

Israel

Anne Greenough

United Kingdom

Ian Griffin

USA

Portia Groening

USA

Dorota Groffik

Poland

Jonathan Gubbay

Canada

Gil Guerra-Junior

Brazil

Hasantha Gunasekera

Australia

Sarika Gupta

India

Samir Gupta

United Kingdom

Gerda-Maria Haas

Germany

Mark Hancock

Australia

Flo Harrison

United Kingdom

Tim Harrison

United Kingdom

Sara Hart

USA

Mohammad Hasan

Canada

Michael Hawkes

Canada

Yasunobu Hayabuchi

Japan

Britt Hedman Ahlström

Sweden

Cheryl Hemingway

United Kingdom

Christinae Hermann

Germany

Ann Hermansson

Sweden

Lise Hestbaek

Denmark

David Hill

Australia

Valeria Hirschler

Argentina

Alan Horn

South Africa

Wendy Hovdestad

Canada

Elizabeth Howard

USA

Philippe Hubert

France

Nils-Olaf Hübner

Germany

Milagros Huerta

USA

Brenda Hughes

New Zealand

Andrea Hunter

Canada

Pertti Huotari

Finland

Colin Hutchison

United Kingdom

Wuh-Liang Hwu

Taiwan

Zubairu Iliyasu

Nigeria

Moshe Ipp

Canada

Brian Irving

USA

Lisa Jackson

USA

Andrew James

Canada

Sarah Jaser

USA

Alessandro Jenkner

Italy

Edwin Jesudason

USA

Eric Jeziorski

France

Yi Ji

China

Fan Jiang

China

Tracy Jirikowic

USA

Chandy John

USA

William Johnson

USA

Teresa Jones

United Kingdom

Saskia Juenger

Germany

Madarina Julia

Indonesia

Beom Cho Jun

South Korea

Karen Kaczynski

USA

Anthony Kafatos

Greece

Paul Kaplowitz

USA

Jordan Kase

USA

David Kaufman

USA

Ilias Kavouras

USA

Akilah Keita

USA

Louise Kelly

USA

Cynthia Kenyon

Canada

Leah Kern

USA

Anuradha Khadilkar

India

Minesh Khashu

United Kingdom

Byung-Eun Kim

USA

Jennifer Kim

USA

Michael Kibet Kiptoo

Kenya

Ian Kitai

Canada

Gil Klinger

Israel

Yiannis Koutedakis

Greece

Sowmya Krishnan

USA

Ashok Kumar

India

Praveen Kumar

India

Chandrakanta Kumar

India

Rashmi Kumar

India

Meena Kumari

United Kingdom

Niraj Kumari

India

Ernesta Kunneke

South Africa

Nyaradzai Edith Kurewa

Zimbabwe

Leora Kuttner

Canada

Sanna Kuusikko

Finland

Kari Kvaerner

Norway

Hannah Ladenhauf

Austria

Harrie Lafeber

Netherlands

Yeur-Hur Lai

Taiwan

Harry Lando

USA

Emily Law

USA

Taiwo Lawoyin

Nigeria

Jean Lawrence

USA

Alfonso Lechuga-Sancho

Spain

Nichung Lee

Taiwan

Matthieu Lenoir

Belgium

Sandrine Leroy

France

Cynthia Leung

Hong Kong

Steven Leuthner

USA

Danielle Levac

Canada

Rong Lin

China

Linda Lindeke

USA

Carsten Lippold

Germany

Robert Locke

USA

Enrico Lombardi

Italy

Abel Lopez-Bermejo

Spain

Lesley Lowes

United Kingdom

Ronit Lubetzky

Israel

Zhong-Cheng Luo

Canada

Empar Lurbe

Spain

Daune Macgregor

Canada

David Mack

Canada

Manu Madhok

USA

Sabine Maguire

United Kingdom

Gamal Mahdi

Canada

Shannon Majowicz

Canada

Imad Makhoul

Israel

Hoda Malaty

USA

Ashwini Mallappa

USA

Marisa Mancini

Brazil

Lindsay Mangham

United Kingdom

Cedric Manlhiot

Canada

Bruno Marino

Italy

Neil Marlow

United Kingdom

Ronella Marom

Israel

Gian Luigi Marseglia

Italy

Marie-Soleil Masse

Canada

Joseph Mathew

India

Amit Mathur

USA

Marja-Leena Mattila

Finland

Gerardo Maupome

USA

Johannes Mayer

Germany

Evan Mayo-Wilson

United Kingdom

Sam Mbulaiteye

USA

Donald McClellan

USA

David McCormick

USA

Brian McCrossan

United Kingdom

Linda McDougal

USA

Brian McFarlin

USA

Patricia McKinley

Canada

Antonio Messineo

Italy

E. Kathryn Miller

USA

Angelo Minucci

Italy

Gerald Mizejewski

USA

Guy Molenaers

Belgium

Diego Moliner-Urdiales

Spain

Lisbeth B Møller

Denmark

Elizabeth Molyneux

Malawi

Paula M.C. Mommersteeg

Netherlands

Paul Monagle

Australia

Guillermo Montes

USA

Ann Montgomery

Canada

Hannah Moore

Australia

Francisco J. Morales-Olivas

Spain

Milena Morano

Italy

Nathalie Morel

France

Jose Manuel Moreno-Villares

Spain

Lindee Morgan

USA

Rintaro Mori

Japan

Andrew Morris

Canada

Sogol Mostoufi-Moab

USA

Theodora Mouratidou

Italy

Marialena Mouzaki

Canada

Anja Mowes

USA

Mwifadhi Mrisho

Tanzania

Michael Msall

USA

Lulu Muhe

Switzerland

Ezekiel Mupere

Uganda

Tasha Murphy

USA

Veera Sekaran Nadarajan

Malaysia

Savithri Nageswaran

USA

Vivek Narendran

USA

Theodore Nash

USA

Girija Natarajan

USA

Gopalakrishnan Netuveli

United Kingdom

David Neubauer

Slovenia

Susan Niermeyer

USA

Saroj Nimkarn

USA

Patricia Nixon

USA

Ellen Nordal

Norway

Deborah O'Connor

Canada

Judith O'Connor

USA

Anke Oenema

Netherlands

Felix Okah

USA

Luciana Oliveira

Brazil

Margaret O'Neil

USA

Eberechukwu Onukwugha

USA

Rianne Oostenbrink

Netherlands

Anne Opavsky

Canada

Charles Opondo

Kenya

Kayode Osungbade

Nigeria

Fabio Paglialonga

Italy

Vassilios Papadakis

Greece

Kathryn Parkinson

United Kingdom

Guido Parodi

Italy

Isela Parra-Rojas

Mexico

Ravi Patel

USA

Sameer Patel

USA

Susmita Pati

USA

Annalisa Patrizi

Italy

Allison Payne

USA

Stephen Pearlman

USA

Carla Pedrosa

Portugal

Ana Belen Peinado Lozano

Spain

Suzanne Penfold

Tanzania

Rafael Perera

United Kingdom

Letizia Perillo

Italy

Martin Petric

Canada

Riccardo Pfister

Switzerland

Shubha Phadke

India

Bob Phillips

United Kingdom

Kong Boo Phua

Singapore

Paul Pianosi

USA

William Pickett

Canada

Russell Pierre

Jamaica

Rajamohanan Pillai

India

Martin Pinquart

Germany

Richard Placzek

Germany

Manuel Pombo Arias

Spain

Kumar Praveen

USA

Michael Preece

United Kingdom

Shahirose Premji

Canada

Elena Proietti

Switzerland

Roman Prymula

Czech Republic

John William Lambert Puntis

United Kingdom

Thanyawee Puthanakit

Thailand

Oliver Quarrell

United Kingdom

Carlo Cosimo Quattrocchi

Italy

Shashi Raj

USA

Francisco Ramos-Gomez

USA

Shripada Rao

Australia

Reeta Rasaily

India

Shantanu Rastogi

USA

Mariusz Ratajczak

USA

John Reilly

United Kingdom

Jon Marc Rhoads

USA

Donato Rigante

Italy

Raili Riikonen

Finland

James Riviello

USA

Joan Robinson

Canada

Domenico Romeo

Italy

Martha Romney

USA

Andrea Ronchi

Italy

Zeev Rosberger

Canada

Sarah Rose

Canada

Galit Rosen

USA

Robert Ruben

USA

Emanuela Russo

Italy

Elizeus Rutebemberwa

Angola

Shane Rutherfurd

New Caledonia

Tzang Ruu-Fen

Namibia

Silvina Ruvinsky

Argentina

C. Anthony Ryan

Ireland

H.P. Sachdev

India

Nicoletta Salerni

Italy

Lucia Salesi

Italy

Jean Pierre Salles

France

Marina Salvadori

Canada

Sixto Sanchez

Peru

Maria Paula Santos

Portugal

Yolanda Sanz

Spain

Carmen Sapienza

USA

Roberto Sassi

Canada

Pieter Sauer

Netherlands

Sonia Saxena

United Kingdom

Sadath Sayeed

USA

Kristin Scheible

USA

Gerd Schmalisch

Germany

Wolf-Peter Schmidt

United Kingdom

Sebastian Schmidt

Germany

Helen Schneider

USA

Sarah Schoen

USA

Salome Scholtens

Netherlands

Jane Scott

Australia

Tomas Seeman

Czech Republic

Angelo Selicorni

Italy

Nick Sevdalis

United Kingdom

Rebecca Shaffer

USA

Prakeshkumar Shah

Canada

Ron Shaoul

Israel

Kevin Short

USA

Michael Siller

USA

Jaivir Singh

India

Ash Singhal

Canada

Linda Slack-Smith

Australia

John Sloper

United Kingdom

Leigh Small

USA

Tom Snijders

United Kingdom

Melissa Snyder

USA

Farin Soleimani

Iran

Neelam Sood

India

Phyllis Speiser

USA

Gemma Spiers

United Kingdom

Jane Squires

USA

Chandrashekhar Sreeramareddy

Nepal

Neeraj Mohan Srivastava

India

Takara Stanley

USA

Lindsay Stead

United Kingdom

Daniela Cristina Stefan

South Africa

John Stefano

USA

Garry Steil

USA

Renato Stein

Brazil

Theodor Stemper

Germany

Jennifer Stinson

Canada

Barbara Stoecker

USA

Matthew Stoll

USA

Ketil Stordal

Norway

Laura Strohmenger

Italy

Veit Sturm

Switzerland

Lynne Sturm

USA

Susan Sullivan-Bolyai

USA

Saradha Suresh

India

Carlos Suslik

Brazil

George A. Syrogiannopoulos

Greece

Andrea Taddio

Italy

Luciana Tanno

Brazil

Karl-Gunter Technau

South Africa

Jeffrey Teckman

USA

Judy Temple

USA

Urmila Thatte

India

Ute Thyen

Germany

Sascha Tierling

Germany

Peter Tilley

Canada

Angela Tobon

Colombia

Lil Tonmyr

Canada

Sara Torretta

Italy

Lisbeth Tranebjaerg

Denmark

Victoria Trenchs

Spain

Daniele Trevisanuto

Italy

Jeanie Tryggestad

USA

Aristotelis Tsiakalos

Greece

David Tudehope

Australia

Mark Turner

United Kingdom

Deborah Tuttle

USA

Sharon Unger

Canada

Ersin Uskun

Turkey

Vytautas Usonis

Lithuania

Christina Valentine

USA

Hassan Vally

Australia

Agnes Van Den Hoogen

Netherlands

Anton Van Kaam

Netherlands

Heleen Van Ommen

Netherlands

Jillon Vander Wal

USA

Maria Inês Varela-Silva

United Kingdom

Scott Veldhuizen

Canada

Milos Veleminsky

Czech Republic

Maximo Vento

Spain

Victor Vila

Spain

Ralf-Peter Vonberg

Germany

Anne Walsh

Australia

Katherine Ward

United Kingdom

Anna Wasilewska

Poland

Jon Watchko

USA

Ralf Weigel

United Kingdom

Carol Weitzman

USA

Li Ming Wen

Australia

William Whitehead

USA

Lesley Wiart

Canada

Jeffrey Wiener

USA

Keoki Williams

USA

Deanne Wilson-Costello

USA

Almut Winterstein

USA

Joshua Wolf

USA

Alastair Woodman

United Kingdom

Charlotte Wright

United Kingdom

James Wynn

USA

Shirley Wyver

Australia

Yoshiyuki Yamada

Japan

Chuan-Zhong Yang

China

Michael Yang

USA

Nan-Ying Yu

Taiwan

Syed Wamique Yusuf

USA

Nabila Zareen

Saudi Arabia

Susan Zeisel

USA

Linjie Zhang

Brazil

Ekhard Ziegler

USA

Frans G. Zitman

Netherlands

Joseph Zorc

USA

## Pre-publication history

The pre-publication history for this paper can be accessed here:

http://www.biomedcentral.com/1471-2431/13/30/prepub

